# Work-related exposures and disorders among physical therapists: experiences and beliefs of professional representatives assessed using a qualitative approach

**DOI:** 10.1186/s12995-016-0147-0

**Published:** 2017-01-07

**Authors:** Maria Girbig, Alice Freiberg, Stefanie Deckert, Diana Druschke, Christian Kopkow, Albert Nienhaus, Andreas Seidler

**Affiliations:** 1 Institute and Policlinic of Occupational and Social Medicine, Faculty of Medicine Carl Gustav Carus, TU Dresden, Fetscherstr 74, 01307 Dresden, Germany; 2 Institute for Health Services Research in Dermatology and Nursing, Hamburg Center for Health Economics, University Hamburg, Hamburg, Germany

**Keywords:** Physical therapist, Occupational diseases, Occupational health, Qualitative research

## Abstract

**Background:**

According to international study results, physical therapists are afflicted with work-related musculoskeletal, psychosocial and dermal disorders as well as infections. The few existing studies in German-speaking regions focus mainly on dermal and psychosocial exposures and resulting complaints. An overview of all relevant work-related exposures and complaints of physical therapists is currently lacking.

We sought to identify work-related exposures based on the subjective experiences and beliefs of physiotherapeutic representatives, in order to identify relevant work-related complaints and diseases. Likewise we aimed to compare the international evidence with the actual situation of physical therapists in Germany.

**Methods:**

Two complementary qualitative approaches were used: 1) a focus group discussion with representatives of professional physiotherapy associations as well as health and safety stakeholders and 2) qualitative semi-structured telephone interviews incorporating currently employed physical therapists. The group discussion was conducted applying a moderation technique, and interviews were analyzed using the content analysis approach by Mayring.

**Results:**

The focus group discussion with five participants and the 40 semi-structured interviews with physical therapists identified comparable results. The main exposures of physiotherapeutic work were considered to be musculoskeletal (e.g., awkward body postures during treatment, patient transfers, passive mobilization), psychosocial (e.g., statutory audit of prescriptions and the associated conflicts with doctors and health insurance providers) and partly dermal and infectious (e.g., wet work and risk of infection) factors. Diseases of the spine, wrist or finger joints, burnout syndrome and infections were mentioned as possible consequences.

**Conclusions:**

The subjective data generated by both groups (focus group discussion and interviews) were comparable and consistent with the current state of research. The results provide new insight regarding work-related exposures and diseases of physical therapists working in Germany. These findings aided the design of a German-wide representative survey of practicing physical therapists.

## Background

Work-related complaints and disorders of physical therapists have been researched consistently since the mid-1980s. International studies of physical therapists identified musculoskeletal diseases, skin diseases and infections as well as mental complaints and diseases to be relevant, with most studies observing increased risks of *musculoskeletal complaints and diseases* involving, in particular, the upper back, lumbar spine, neck, shoulders, wrist/hands, knees and thumbs [1–7]. Studies focusing on *mental complaints*, also found a low to moderately increased prevalence of burnout syndrome among physical therapists [8–11]. Concerning *work-related skin diseases* of physical therapists, only single reports [12–14] and an overview about potentially harmful substances [15] exist in the literature. However, contact with patients’ open wounds (e.g., risk of hepatitis C, hepatitis B or HIV infection) or with infectious patients (e.g., tuberculosis and Methicillin-Resistant Staphylococcus aureus (MRSA)) is associated with an increased *risk of infection* [16, 17].

In Germany, scientific research of workload among non-physician health care professionals has focused mainly on caregivers and nurses [18–20], although physical therapists, with currently about 228 000 employees in Germany [21], represent a significant occupational group within the health care sector. The few existing articles focusing on physical therapists in Germany indicate that the physiotherapeutic work is associated with psychosocial [22, 23] and dermal exposures [24] as well as diseases [15]. However, a comprehensive national or international overview of all relevant work-related complaints and diseases among physical therapists is missing to date.

The main objectives of this work are, to give a first overview of perceived work-related exposures and disorders among physical therapists in Germany from the perspective of experts, and to determine if the results are consistent with international research on this the topic.

## Methods

Using an exploratory approach, two qualitative methods with purposive sampling strategies were applied simultaneously to identify experiences, attitudes and knowledge of professionals regarding physiotherapeutic work-factors (including workload and resources) and work-related complaints and diseases as well as possible associations between them:

1. A focus group discussion with experts of German professional physiotherapy associations as well as health and safety stakeholders,
2. Semi-structured interviews with physical therapists currently working in Germany.

Subsequently, the results of both methods were analyzed and compared.

The study is reported according to the consolidated criteria for reporting qualitative studies (COREQ) [25].

### Focus group discussion

The aim of this approach was to assess the professional policy perspective. Therefore, experts from German professional physiotherapy associations as well as health and safety stakeholders were included, and a focus group discussion was conducted to utilize group dynamic processes and the participants’ discussion as a source of knowledge [26]. In addition, this method allows the direct representation of information about similar and different opinions of participants [27] and reasons for individual statements [28].

#### Setting and participants

One representative of each German professional physiotherapy association *(Bundesverband selbstständiger Physiotherapeuten (IFK) e. V.; Physiotherapieverband e. V. (VDB); Verband Physikalische Therapie (VPT); Deutscher Verband für Physiotherapie (ZVK) e. V.)* and one representative of the Institution for Statutory Accident Insurance and Prevention in the Health and Welfare Services *(Berufsgenossenschaft für Gesundheitsdienst und Wohlfahrtspflege, BGW)* were invited to participate in the focus group discussion. The focus group discussion was conducted at the Institute and Policlinic of Occupational and Social Medicine, Technical University Dresden.

#### Material and analysis

An a priori defined semi-standardized guideline, based on the international state of research on work-related exposures (with reference to the work area and/or employment status) and diseases of physical therapists was utilized. To achieve a structured and consensus based discussion result, the whole focus group discussion was conducted by applying the moderation method [29]. Two moderators (scientists with expertise in physical therapy and psychology) (M.G. and D.D.) involved all participants equally and were responsible for a structured group discussion. First, participants were asked about individual perceptions concerning typical work-related exposures. The second part of the discussion contained questions regarding characteristics of work-related complaints and diseases, and the third part focused on perceptions of work-related resources. The guideline was used by the moderators as orientation for the course of the discussion, nevertheless, thematic digressions were allowed.

Flipcharts and magnet boards were used to illustrate consensus results. The discussion was recorded visually by two persons and the results were documented with photos. Group discussion results were summarized based on a cluster building process of the work-related exposures, complaints and diseases as well as resources. In addition, the final protocol was mailed to the participants for their acceptance.

### Semi-structured interviews

Semi-structured interviews were performed to identify physiotherapeutic work-related exposures as well as main complaints and diseases from the perspective of working physical therapists themselves.

#### Setting and participants

Interviewees were selected from different employment settings to represent as many work-related facets as possible, and to obtain a heterogeneous group of German physical therapists. We attempted to include professional physical therapists with as many combinations of the following criteria as possible:

- gender,
- region: north, south, east and west of Germany,
- professional experience (entrants and professionally experienced) and
- work area: hospital/rehabilitation (inpatient) and ambulance (outpatient).

Selection also guaranteed that physical therapists working in different disciplines (orthopedics/surgical ward; neurology; internal medicine and other) were represented. The recruitment was realized using the Yellow Pages®, where businessmen and -women in Germany – including self-employed physical therapists – offer their services. A list of all physiotherapy practices from three German federal states (Saxony, Baden- Wurttemberg and Berlin) was generated to include different regions. These therapists were contacted per email and/or telephone and asked to participate in our study. Physical therapists were interviewed at their workplace or home by telephone.

#### Material and analysis

Standardized semi-structured telephone interviews were pilot-tested and conducted using an interview guideline developed by two physical therapists and a psychologist (M.G., C.K., D.D.) (Table 1). The guideline was based on the current international state of research regarding work-related exposures, complaints and diseases of physical therapists. The guideline provided orientation for the interviewer, but still allowed for thematic digressions. Demographic and occupational data were also collected from all participants. Basically, the questions were open-ended to promote as unaffected an exchange of opinions and attitudes as possible. In addition, minor questions were provided to be used when detailed explanations of the initial questions were necessary or when only parts of the corresponding subject area were addressed. Data collection continued until the research team agreed that no new themes were being generated and data saturation had been achieved. Each semi-structured interview was recorded digitally, transcribed and anonymized. Consent was obtained verbally prior to the start of interviews. Interviews were analyzed using Mayrings’ qualitative content analysis [30]. To achieve an understanding of the data, a coding system was developed for categorizing participants’ answers, which allowed for the reduction of statements and detecting groups of answers. A mixed inductive-deductive approach was used, meaning that, on the one hand, already existing international knowledge was integrated; on the other hand, new categories were formed based on the interview material. The finalized coding frame was systematically applied to all transcripts by one data coder using quali.xls [31]. Citations were translated into the English language by a native speaker for the purpose of publication.

**Table 1:** Interview guideline for semi-structured telephone interviews

**1.**What does a typical day in your practice/on your station look like?
Important points that should be answered within this question:
▪ What are your main activities at work?
▪ How many patients do you treat every day on average?
▪ How much time do you have on average for one treatment?
▪ How many breaks do you take per day? How long are these?
▪ Do you have additional time slots for scheduling and documentation tasks?
▪ Is tidying and cleaning a part of your responsibilities? If so, do you do this within working hours?
**2.**Do you have the feeling that your work as physiotherapist has an impact on your health in any way (positive or negative)?
Important points that should be answered within this question:
▪ In what area of your health do you think your work has a special influence and how does this manifest itself? – With respect to e.g.,: physical health; mental health; skin complaints/disorders or infections
▪ Do you think that the physiotherapy knowledge and the work in this area are beneficial to your health?
▪ Do you have complaints/diseases, which are associated with your work as a physiotherapist?
**3.**What factors (activities, circumstances etc.) do you see in your work as physiotherapist as particularly stressful?
▪ If symptoms/diseases exist that originate from the physiotherapy work:
- What do you think are the specific working conditions/factors that have led to these complaints/diseases?
▪ How do you deal with the designated strain? What do you do for compensation? – e.g., leisure activities (sports etc.) or compensation with additives within the stressful activity
**4.**How do you feel when you go to work in the morning or come back home in the evening?
Important points that should be answered within this question:
▪ Do you look forward to your work and the contact with patients?
▪ What do you like/do you not like about your job?
▪ With your current knowledge, would you absolve qualification as a physiotherapist once again?
▪ Have you ever thought about leaving your job? Why?
▪ Are you planning to attend any further training/study? What?

Each participant of both qualitative approaches was offered the results of the study.

## Results

The focus group discussion [32] involved five participants (see aforementioned affiliations) aged between 30 and 73 years. The age of the 40 interviewed working physical therapists ranged between 25 and 56 years. Participant characteristics are summarized in Table 2. The focus group discussion continued four hours and the interviews lasted an average of 35 min.

**Table 2:** Demographic characteristics of participants

	Focus group discussion	Semi-structured interviews
Total respondents (number)	5	40
Sex – Female (number (%))	4 (80%)	22 (55%)
Age – Range (years)	30-73	25-56
Clinical Setting	N.A.	
Inpatient (number (%))		5 (12.5%)
Outpatient (number (%))		25 (62.5%)
Both (number (%))		10 (25.0%)
Employment status^a^	N.A.	
Full time (≥39 h/ week)		24 (60.0%)
Part time		15 (37.5%)
Professional experience^b^	N.A.	
1 – 10 years (number (%))		12 (30.0%)
11 – 20 years (number (%))		14 (35.0%)
21 – 30 years (number (%))		5 (12.5%)
31 – 40 years (number (%))		4 (10.0%)

Notes: N.A. not applicable, ^a^missing value of one person, ^b^missing value of five persons

The results of both qualitative approaches were first considered separately and subsequently compared.

### Focus group discussion

#### Work-related exposures

**Fig. 1:**
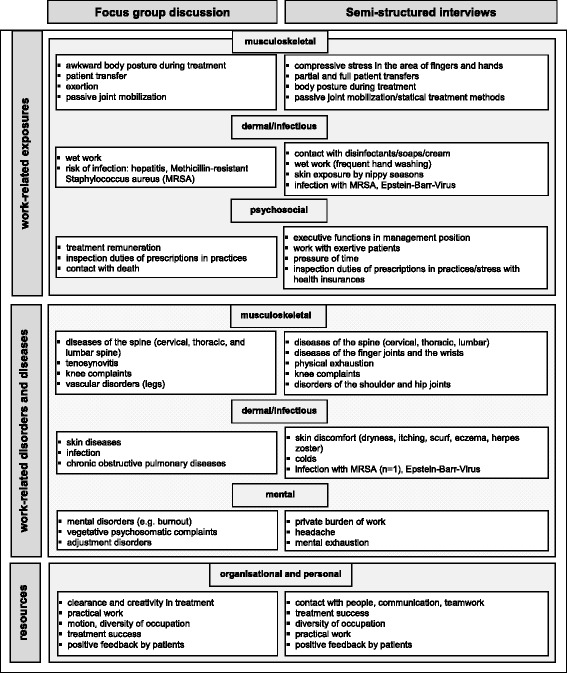
Complementary juxtaposition of results of both qualitative approaches

Musculoskeletal and psychosocial factors were identified as the two main exposure categories afflicting German physical therapists (Fig. 1). Especially awkward postures during treatments, patient transfers, exertion and passive joint mobilizations were named as the most relevant *musculoskeletal exposures* of this profession. Participants also believed that underpayment, obligations to check prescriptions and dealing with death are the most important *psychosocial exposures* faced by physical therapists.

Based on the current state of research, the moderators pointed out additional factors requiring discussion. The experts were asked to vote on whether they believed dermal, infectious or physical working conditions might play a role within physiotherapeutic work. In this context, *dermal exposures*, especially wet work and risk of infections, were appraised as important for the occupation. Even though work-related *infections* are more frequent among caregivers and nurses, they are also considered relevant for physical therapists. Regarding physical work factors related to shortwave and microwave treatments, participants were discordant on how to classify these special kinds of work-related factors. The reason for inconsistent opinions was the fact that physical therapists in Eastern Germany applied elements of electrotherapy more often than their colleagues in Western Germany. As a consequence, thermic factors like burns and scalding were unanimously voted as being partially relevant for physical therapists.

According to the predefined consensus, participants discussed the relevance of the named work-related exposures of physical therapists in the context of work setting and employment status. As a result, the existence of physical exposures seems to be particularly associated with the respective work area. In contrast, psychosocial exposures were more strongly associated with employment status. Thus, physical therapists in the outpatient sector are especially confronted with increased physical exertion, while the self-employed are particularly exposed to psychosocial strain due to economic stress and existential fears.

#### Work-related complaints and diseases

Following the discussion of work-related exposures, focus group participants discussed if complaints and diseases (including occupational diseases) could possibly be connected with the pertinent exposures. According to the experts, among the diseases included in the German list of occupational diseases, the following are potentially relevant for physical therapists:

- Severe or recurrent skin diseases (No. 5101), e.g., by use of massage lotion, salve or gloves and wet work;
- Disc-related diseases of the lumbar spine (No. 2108), e.g., by patient transfer (including partial body transfer);
- Infectious diseases (No. 3101) and Obstructive diseases of the respiratory tract (No. 4301 & 4302) e.g., by working with ill patients and allergenic substances (especially in inpatient and/or elderly care).

In addition, other complaints/diseases were named which might be (in part) due to the work as a physical therapists. These include further complaints of the spine (cervical spine and thoracic spine), tenosynovitis, knee complaints and circulatory disorders (e.g., varices). Within the area of mental complaints and disorders, burnout syndrome, vegetative-psychosomatic complaints and adjustment disorders were proposed as most important.

#### Resources

The experts pointed out that the physiotherapeutic work also includes health promoting aspects; consequently, resources were considered. In particular, the participants highlighted the independence and creativity involved in selecting and implementing treatments, practical work, occupational diversity, treatment success and positive feedback from patients as having a beneficial effect on the health of physical therapists.

### Semi-structured interviews

The results of the interviews are listed below according to frequency of nomination. In addition, some particularly concise quotations of individual people are presented.

#### Work-related exposures

The most commonly reported *musculoskeletal exposures* were pressure loads in the area of fingers and hands, partial and full patient transfers, awkward body postures during treatments, non-ergonomic working conditions and prolonged standing at the treatment table.

*“Knowing what I know now, I wouldn’t choose the profession again – because of the physical exertion.”*

Regarding psychosocial conditions, the main *psychosocial exposures* considered were executive functions (in management positions), working with difficult patients, time pressure and problems with the administrative processing of prescriptions in private-practices (stress with health insurance providers).

*“It bothers me when patients are rude.”*

*”I don’t like being treated like a “waste bin” for some patients.”*

*”I don’t like the trouble with the health insurance companies- incorrectly completed prescriptions, no money for services performed, messed around with like a dancing bear.”*

*“Due to prescriptions being returned by a particular health insurance company… I regularly explode on the phone.”*

The most frequently mentioned *dermal exposures* were contact with disinfectants, soaps and creams and frequent hand washing (wet work). Contact with infected patients was rarely considered by the respondents as an exposure. In this regard only infections with Methicillin-Resistant Staphylococcus aureus (MRSA) and the Epstein-Barr Virus were specified.

#### Work-related complaints and diseases

The main *musculoskeletal complaint* named by the interviewees was discomfort of the spine. Eight of these therapists could not further specify their problems. Among the remaining therapists, complaints were located in the cervical spine, lumbar spine and thoracic spine. Other frequently affected body areas mentioned in the interviews were wrist and finger joints, knees, shoulders and hips. Some of the respondents perceived physical exhaustion at the end of the day. Slightly more than a quarter of the consulted physical therapists had not yet experienced any work-related impacts on their physical health or well-being. According to the respondents, work-related *psychosocial exposures* are particularly transferred into the private life, and in this regard, were associated with headache (*n* = 4) and mental fatigue.

*”At the end of the day I have the feeling that my brain is full of knots.”*

*”I don’t want to talk in the evening.”*

*”To me, the psychological burden is greater than the physical.”*

Work-related *complaints of the skin*, such as skin dryness, itching, peeling, eczema and herpes zoster were rated by one quarter of the physical therapists as relevant to their work. With respect to possible *risks of infection*, two physical therapists working with outpatients reported that they suffer from influenza infection more often due to work. Two others had already undergone an infection with MRSA and Epstein-Barr virus, respectively.

#### Resources

The most frequently named work-related resources by the interviewed therapists were contact with people, communication and teamwork. In addition, treatment success, occupational diversity, the practical work as well as positive feedback from the patients were considered as benefits.

*”If the payment would be better, it would be almost a dream job”*

The subjective data of both groups of professional representatives were assimilable and consistent with the current state of research. A comparison of the main results of both qualitative approaches can be found in Fig. 1.

Awkward body postures during treatments, patient transfers and passive joint mobilization were specified as the *main work-related exposures* of musculoskeletal complaints and diseases. Professionals also often mentioned stress due to compressive load from working tasks on fingers and hands. In the area of work-related psychosocial stress factors, particularly the existing statutory audit of prescriptions and the associated conflicts with physicians and health insurance providers was considered as a burden. Wet work and the risk of infection with methicillin-resistant Staphylococcus aureus (MRSA) were considered to be the main dermal and infectious problems by both groups. W*ork-related complaints and diseases* in the whole area of the spine were described by both groups as the main existing musculoskeletal disorders. Results of semi-structured interviews additionally indicated that complaints of the fingers and wrists as well as general physical exhaustion are potential problems. Both, the political experts and the professionals named skin disorders and diseases as common work-related problems. The participants of the focus group mentioned mental and psychosomatic symptoms as well as autonomic adjustment disorders as the foremost occurring mental disorders. The working professionals named headache, mental fatigue and private strain because of their occupation as particularly important. Both political experts and working professionals identified the practical work of physical therapists, the existing scope for professional development, the diversity of activities, the positive feedback from patients and the possibilities of teamwork, as essential *resources of the occupation*.

## Discussion

Using the two qualitative approaches presented (focus group discussion and semi-structured interviews), the entire spectrum of work-related exposures and diseases of physical therapists in Germany was investigated for the first time. According to the results, work-related musculoskeletal, mental, dermal and infectious complaints and diseases may compromise the health of physical therapists. Basically the specifications of both qualitative approaches are very similar or complement each other very well (see Fig. 1). However – particularly in the field of psychosocial exposures - the prioritization of the named factors appears to be different (the order of the points listed in the boxes indicates their priority). The participants of the focus group discussion were more concerned with current political topics such as remuneration of treatment and inspection duties of prescriptions in practices. In comparison, the participants of the interviews (mostly physical therapists in outpatient settings) were more focused on the strain of the daily practical work as physical therapist, like the work with difficult patients and time pressure. We think these differences are due to the diverse current working activities of the participants (focus group discussion: mostly management positions/interviews: practical working German physical therapists) and the resulting differences in the importance of specific stress areas.

There were also slight differences in the data on mental disorders and diseases. For example the burnout syndrome as a possible mental disorder of physical therapists was – consistent with existing national [22] and international studies [33–37] – specifically mentioned within the focus group discussion (not by the interviewees). This, together with the other data in this area allows the assumption that the participants of the focus group discussion were more concerned with this field of study, or had more points of contact with the issue of work-related disorders/diseases in general (one participant was a representative of the Institution for Statutory Accident Insurance and Prevention in the Health and Welfare Services). Likewise, the results provide the first evidence for the transferability of international research on work-related exposures and disorders to physical therapists working in Germany.

Until now, neither national nor comparable international qualitative studies reflecting the experiences and beliefs of physiotherapeutic experts comprehensively examined all important work-related exposures, complaints and diseases. For the first time, the present study summarizes all work-related exposures and health outcomes of physical therapists in Germany considered relevant by experts. The few existing international qualitative studies focused only on musculoskeletal exposures and complaints as central themes [38–40]. German-speaking studies with qualitative approaches dealing with work-related exposures or complaints of physical therapists mainly focused on psychosocial exposures and diseases [23, 41] or job satisfaction in general [42].

Within our study new aspects were specified in the field of psychosocial exposure of physical therapists which have not been mentioned in the international literature so far, for example obligations to check prescriptions and conflicts with health insurance providers and/or physicians. In particular, self-employed physical therapists reported being concerned by economic stress and existential fears while physical therapists working as employees are often confronted with underpayment, time pressure and significant exertion. These issues appear to be typical for the situation of German physical therapists. For example, within the German healthcare system, patients in need of physiotherapy services have to first obtain a prescription for the treatment from a physician (i.e., patients do not have direct access to physiotherapy like in other countries, such as Australia, the Netherlands, Sweden, Norway, Great Britain and Canada etc.). These prescriptions will be only acknowledged (and paid) by the according health insurance if all data are complete and filled in correctly. The verification of the data is the responsibility of the attending physical therapist. If there are any problems, the therapist (or the patient) has to deal with the treating physician or the health insurance. This requires considerable administrative effort and can lead to existential fears in the affected practices. The remuneration of physiotherapy services by the health insurances in Germany is considered to be too low. This results in the frequently discussed low payment of physical therapists (especially in East Germany), and in an increased turnover rate of professionals [23, 43].

Concerning the most important musculoskeletal exposures (e.g., patient transfer, awkward body postures and passive joint mobilization) as well as the body areas mainly affected by musculoskeletal complaints (spine, fingers and wrists as well as knees), the findings of the present study are consistent with the current state of international research described above [1–7]. Occupational skin diseases (specific diagnoses were not mentioned) and corresponding exposures within the physiotherapeutic work were named, but considered to be less relevant by both expert groups. This is in line with the current state of international research but in contrast to the fact that work-related skin diseases (No. 5101) were the most reported (n = 311) and recognized (n = 15) occupational diseases for physical therapists in Germany according to data of the German Institution for Statutory Accident Insurance and Prevention in the Health and Welfare Services (BGW) in 2014 (personal information of the BGW, 2015). The discrepancy might be a result of participant selection, and subjects with only few or no skin problems may have been selected by chance or subjects with skin problems might have been on sick leave due to their skin diseases. Subsequently, this may have yielded less consideration of work-related skin diseases within the focus group discussion as well as the interviews. However, the recognition incidence of musculoskeletal occupational diseases in Germany is generally low. Therefore, the recognition of occupational diseases is not necessarily directly associated with the actual prevalence of these disorders among professionals in this field. International studies of infections among physical therapists are rare and focus mainly on hepatitis B infections [16] and tuberculosis [17]. In contrast, infections with MRSA and Epstein-Barr-Virus were named in our assessment.

The present study addresses for the first time the entire spectrum of possible work-related exposures and complaints as well as diseases of physical therapists in Germany. A qualitative approach was chosen to gain a first impression of this research area and to screen to what extent the results of past international research applies to Germany.

The greatest *strength* of this study is the use of two different qualitative methods. This enables the different perspectives of two groups of physiotherapeutic profession experts to be captured. Through the focus group discussion, a wide spectrum of knowledge and opinions could be gained because a representative of each existing professional physiotherapy association in Germany participated. In addition, the study sample of the semi-structured interviews was relatively large and the participants were recruited from a wide geographical area of Germany with varying professional backgrounds and experiences. This was very important because the work as physical therapist in Germany varies greatly depending on the work area (inpatient or outpatient), discipline (e.g., surgery, neurology, internal medicine etc.) and acquired advanced training (e.g., manual therapy, manual lymphatic drainage, sports physical therapy etc.).

General *limitations* of qualitative research, which also pertain to this study, are the missing generalizability of the knowledge gained to other people or settings and the possibility that the results were influenced by the researcher (data collection and categorization) [44]. The results of the present study are consistent with the international literature, suggesting these limitations did not greatly impact the study results. One additional potential limitation of focus group discussions can be that the group itself may influence the produced data, and a classical problem is the influence of the group interaction on the individual contribution of each participant in the group discussion [45]. However, any resulting distortion of results could be compensated by the individual interviews.

## Conclusions

The results of our study indicate that physical therapists in Germany generally have an increased risk of musculoskeletal and psychosocial exposures, and to a lesser degree dermal and infectious exposures. Furthermore, a possible association between musculoskeletal and mental complaints was indicated. To verify these potential relationships, a representative study of physical therapists is necessary. For this reason, the results of our described exploratory method was used to design a comprehensive study to investigate the detected variety of work-related exposures, complaints and diseases [46].

## Abbreviations

BGW: Berufsgenossenschaft für Gesundheitsdienst und Wohlfahrtspflege
COREQ: Consolidated criteria for reporting qualitative studies
IFK: Bundesverband selbstständiger Physiotherapeuten
MRSA: Methicillin-Resistant Staphylococcus aureus
VDB: Physiotherapieverband e. V.
VPT: Verband Physikalische Therapie
ZVK: Deutscher Verband für Physiotherapie e. V.
